# Roles of PI3K/Akt and c-Jun Signaling Pathways in Human Papillomavirus Type 16 Oncoprotein-Induced HIF-1α, VEGF, and IL-8 Expression and *In Vitro* Angiogenesis in Non-Small Cell Lung Cancer Cells

**DOI:** 10.1371/journal.pone.0103440

**Published:** 2014-07-24

**Authors:** Erying Zhang, Xiaowei Feng, Fei Liu, Peihua Zhang, Jie Liang, Xudong Tang

**Affiliations:** 1 Institute of Biochemistry and Molecular Biology, Guangdong Medical College, Zhanjiang, Guangdong, China; 2 Guangdong Provincial Key Laboratory of Medical Molecular Diagnostics, Guangdong Medical College, Zhanjiang, Guangdong, China; 3 Institute of Plastic Surgery, Affiliated Hospital of Guangdong Medical College, Zhanjiang, Guangdong, China; University of South Alabama, United States of America

## Abstract

**Background and Objectives:**

Human papillomavirus (HPV)-16 infection may be related to non-smoking associated lung cancer. Our previous studies have found that HPV-16 oncoproteins promoted angiogenesis *via* enhancing hypoxia-inducible factor-1α (HIF-1α), vascular endothelial growth factor (VEGF), and interleukin-8 (IL-8) expression in non-small cell lung cancer (NSCLC) cells. In this study, we further investigated the roles of PI3K/Akt and c-Jun signaling pathways in it.

**Methods:**

Human NSCLC cell lines, A549 and NCI-H460, were stably transfected with pEGFP-16 E6 or E7 plasmids. Western blotting was performed to analyze the expression of HIF-1α, p-Akt, p-P70S6K, p-P85S6K, p-mTOR, p-JNK, and p-c-Jun proteins. VEGF and IL-8 protein secretion and mRNA levels were determined by ELISA and Real-time PCR, respectively. The *in* *vitro* angiogenesis was observed by human umbilical vein endothelial cells (HUVECs) tube formation assay. Co-immunoprecipitation was performed to analyze the interaction between c-Jun and HIF-1α.

**Results:**

HPV-16 E6 and E7 oncoproteins promoted the activation of Akt, P70S6K, P85S6K, mTOR, JNK, and c-Jun. LY294002, a PI3K inhibitor, inhibited HPV-16 oncoprotein-induced activation of Akt, P70S6K, and P85S6K, expression of HIF-1α, VEGF, and IL-8, and *in* *vitro* angiogenesis. c-Jun knockdown by specific siRNA abolished HPV-16 oncoprotein-induced HIF-1α, VEGF, and IL-8 expression and *in* *vitro* angiogenesis. Additionally, HPV-16 oncoproteins promoted HIF-1α protein stability *via* blocking proteasome degradation pathway, but c-Jun knockdown abrogated this effect. Furthermore, HPV-16 oncoproteins increased the quantity of c-Jun binding to HIF-1α.

**Conclusions:**

PI3K/Akt signaling pathway and c-Jun are involved in HPV-16 oncoprotein-induced HIF-1α, VEGF, and IL-8 expression and *in* *vitro* angiogenesis. Moreover, HPV-16 oncoproteins promoted HIF-1α protein stability possibly through enhancing the interaction between c-Jun and HIF-1α, thus making a contribution to angiogenesis in NSCLC cells.

## Introduction

Lung cancer is the leading cause of cancer-related deaths worldwide, and mortality rates continue to increase among older women with lung cancer in many countries [1]. Non-small cell lung cancer (NSCLC) comprises the majority of lung cancer. Cigarette smoking is considered the major risk factor for NSCLC. However, approximately 25% of all lung cancer cases have been observed in never-smokers [2], [3]. Moreover, it was reported that there are different epidemiologic evidences, clinicopathologic features, and survival rates between ever-smoking and never-smoking NSCLC patients [4]–[6], implying that never-smoking NSCLC might be a different disease and have different risk factors [5], [7]. Therefore, other non-smoking risk factors might contribute to never-smoking NSCLC.

In the early 1980s, Syrjanen first suggested the possibility of human papillomavirus (HPV) involvement in bronchial squamous cell carcinoma [8]. Afterwards, a growing body of epidemiological evidence from different countries has shown that the positive rate of high-risk HPV-16/18 DNA and *E6* and *E7* oncogenes in NSCLC was much higher than that in benign lung neoplasms [9]–[16], wherein HPV-16 was the most prevalent HPV genotype with frequent *E6*/*E7* oncogene expression [10], [13], [16]. It is worth noting that the prevalence of HPV infection in clinical specimens of bronchial carcinomas is widely divergent in different geographic regions and histological tissue types, ranged from 0.0 to 100% [17], [18]. But high-risk HPV infection, especially HPV-16, in NSCLC patients has a higher prevalence in Asia, especially in China [9], [11], [12], [15]. Recently, high levels of IgG against HPV-16 and 18 E7 in 16% of NSCLC patients were also detected [18]. With the progress of the studies, high-risk HPV infection has been proposed as a potential cause for NSCLC [17], [18].

Angiogenesis is required for invasive tumor growth and metastasis and plays an important role in the development and progression of cancer including NSCLC [19]–[21]. Angiogenesis, inflammation, and coagulation markers were found to increase in NSCLC patients [21]. Increased levels of vascular endothelial growth factor (VEGF), a key angiogenic factor, correlated with a poor prognosis in NSCLC patients [21], [22]. Hypoxia inducible factor-1α (HIF-1α) was suggested to be an important upstream molecule mediating VEGF expression and angiogenesis. It was reported that there was an association of HIF-1α polymorphisms with susceptibility to NSCLC [23]. Additionally, interleukin-8 (IL-8), a pro-inflammatory chemokine, has also been found to be associated with NSCLC risk [24], [25]. Therefore, HIF-1α, VEGF, and IL-8 play key roles in the development of NSCLC. Interestingly, our previous study has demonstrated that HPV-16 E6 and E7 oncoproteins promoted HIF-1α protein accumulation and HIF-1α-dependent VEGF and IL-8 expression in NSCLC cells [26]. However, the underlying mechanisms by which HPV-16 oncoproteins enhanced HIF-1α, VEGF, and IL-8 expression in NSCLC cells remain unclear.

Previous studies have demonstrated that multiple signaling pathways including phosphoinositide 3-kinase (PI3K)/Akt/mammalian target of rapamycin (mTOR) and mitogen-activated protein kinase (MAPK) signaling pathways mediate HIF-1α and VEGF expression induced by hypoxia or insulin-like growth factor-1 (IGF-1) in various cancer cells [27]–[30]. PI3K/Akt/mTOR signaling pathway has been well characterized and recognized to play essential roles in lung cancer cell proliferation and survival [31]. There are three major MAPK signaling pathways, namely, signal-regulated kinase (ERK), c-Jun N-terminal kinase (JNK), and p38 MAPK pathways. Targets of JNK pathway include the activator protein 1 (AP-1) group of transcription factors, such as Jun. c-Jun contributes to transformation and cancer development and JNK activation has been demonstrated to be involved in the control of the tumor-initiating capacity of NSCLC cells [32]. Therefore, PI3K/Akt/mTOR and MAPK signaling pathways play crucial roles in the initiation and development in NSCLC. Moreover, our previous studies have demonstrated that PI3K/Akt and ERK1/2 signaling pathways were involved in HPV-16 E6- and E7-induced HIF-1α protein accumulation in C-33A and HeLa cervical cancer cell lines [33]. However, the roles of PI3K/Akt and MAPK signaling pathways in HPV-16 oncoprotein-induced HIF-1α, VEGF, and IL-8 expression in NSCLC cells have not been reported.

In this study, we investigated the roles of PI3K/Akt and JNK/c-Jun signaling pathways in HIF-1α, VEGF, and IL-8 expression, and *in* *vitro* angiogenesis induced by HPV-16 E6 and E7 oncoproteins in NSCLC cells. We found for the first time to our knowledge that PI3K/Akt signaling pathway and c-Jun were involved in HPV-16 E6- and E7-induced HIF-1α, VEGF, and IL-8 expression in NSCLC cells, leading to angiogenesis *in* *vitro*. HPV-16 E6 and E7 oncoproteins promoted HIF-1α protein stability through enhancing the interaction between HIF-1α and c-Jun, thus triggering HIF-1α-mediated angiogenesis in NSCLC cells.

## Materials and Methods

### Regents

Transfection reagent (Lipofectamine 2000) was obtained from Invitrogen Corporation (Carlsbad, CA). *In vitro* angiogenesis assay kit (ECM625) was from Millipore (Temecula, CA, USA). Mouse anti-human HIF-1α monoclonal antibody was from BD Transduction Laboratories (San Diego, CA, USA). Mouse anti-human β-actin antibody was purchased from Beyotime Biotechnology Corporation, Shanghai (Shanghai, China). One Step SYBR PrimeScript RT-PCR (No.DRR086A) was purchased from TaKaRa Biotechnology (Dalian) Co., LTD (Dalian, China). Total and phosphorylated Akt (Ser^473^), P70S6K (Thr^389^ and Thr^421^), P85S6K, mTOR (Ser^2481^), JNK (Thr^183^/Tyr^185^), and c-Jun (Ser^63^) antibodies were purchased from Cell Signaling Technology (Beverly, MA, USA). Anti-VHL antibody was from Santa Cruz Biotechnology (Santa Cruz, CA, USA). G418 was from Sigma (St. Louis, MO, USA). MG132, Cycloheximide (CHX), LY294002, SP6002125, normal rabbit IgG, protein A garose beads, and Immunol Fluorence Staining Kit were from Beyotime Biotechnology Corporation, Shanghai (Shanghai, China). Human VEGF and IL-8 Enzyme-linked immunosorbent assay (ELISA) reagent kits were purchased from Wuhan Boster Bio-engineering limited company (Wuhan, China).

### Cell lines and Cell Culture

Human NSCLC cell line A549 (adenocarcinoma cell line) and human umbilical vein endothelial cells (HUVECs) were obtained from American Type Culture Collection (ATCC; Rockville, MD). Human NSCLC cell line NCI-H460 (a large cell lung cancer cell line) was purchased from Chinese Academy of Sciences Cell Bank of Type Culture Collection (CBTCCCAS; Shanghai, China). All cells were cultured in RPMI-1640 media supplemented with 10% FBS, penicillin (100 U/mL) and streptomycin (100 µg/mL) (Invitrogen). All cultures were maintained at 37°C in a humidified atmosphere with 5% CO_2_.

### Stable transfection and establishment of stable-transfected cells

The method was as described previously [34]. Briefly, A549 and NCI-H460 cells at 70% to 80% confluence were respectively transfected with pEGFP empty vector, pEGFP-16 E6, E6 mutant (E6m), E7, and E7 mutant (E7m) plasmids using lipofectamine 2000 (Invitrogen) according to the manufacturer’s instructions. G418 (400 µg/mL in RPMI 1640) combined with flow cytometry was used to screen transfected cells. The expression of HPV-16 E6 or E7 oncoprotein was detected by Western blotting every other week [34].

### RNA interference

The target sequences of RNA interference (RNAi) against c-Jun were previously confirmed [35] and synthesized by Shanghai GenePharma Co., Ltd (Shanghai, China). The sequences of sense strand-directed siRNA against c-Jun are as follows: c-Jun siRNA-1(Si-1) 5′-GAUGGAAACAGC CUUCUAUTT -3′; c-Jun siRNA-2(Si-2) 5′-CCUCAGCAACUUCAACCCATT-3′ [Genbank:NC_000001.10] [35]. HPV-16 E6 or E7-transfected NSCLC cells were cultured in 35 mm plates with growth media without antibiotics. The cells were transiently co-transfected for 4 h with c-Jun siRNA or non-specific control siRNA (NS-siRNA) *via* lipofectamine 2000 (Invitrogen) according to the manufacturer’s instructions. After 48 h incubation at 37°C, the cells were harvested and subjected to Western blot analysis, and the conditioned media were used to ELISA and *in* *vitro* angiogenesis assay.

### Protein extraction and Western blotting

The method was as described previously [26]. Briefly, total proteins were extracted from treated and untreated cells with lysis buffer containing 20 mmol/L Tris (pH 7.5), 1 mmol/L EDTA, 150 mmol/L NaCl, 1% Triton X-100, leupeptin, sodium pyrophosphate, EDTA, leupeptin, β-glycerophoshpate, Na_3_VO_4,_ phenylmethylsulfphonylfluoride (PMSF), and complete protease inhibitor cocktail (Sigma), followed by incubation at 4°C for 1 h. The lysates were ultra-sonicated and centrifuged at 12,000×*g* for 10 min. Protein concentrations were determined by BCA methods. The proteins were separated on 10% polyacrylamide-SDS gel and electro-blotted onto polyvinylidene difluoride (PVDF) membrane. After blocking with TBS/5% skim milk, the membrane was incubated overnight at 4°C with primary antibodies, followed by incubation with HRP-conjugated secondary antibodies. As a loading control, the blots were stripped and re-probed with anti-β-actin antibody.

### Co-immunoprecipitation

The cells were lysed with IP lysis buffer containing 20 mmol/L Tris (pH 7.5), 150 mmol/L NaCl, 1% Triton X-100, sodium pyrophosphate, β-glycerophosphate, EDTA, Na_3_VO_4_, and leupeptin, followed by centrifugation. Total lysate (0.5 mg) was incubated with 2 µg anti-c-Jun antibody overnight at 4°C. The lysate incubated with normal rabbit IgG served as a negative control. Protein A agarose (40 µL) was added into the mixture and incubated with agitation for an additional 4 h at 4°C. The beads were washed thrice with cell lysis buffer for 10 min each time. The precipitants were dissolved with the SDS loading buffer for the analysis of HIF-1α and c-Jun protein by Western blotting.

### Cell immunofluorescence

The cells (2×10^6^) were seeded onto coverslips in 6-well plates. 12 h later, the cells were rinsed with PBS, fixed with immunostaining fix solution overnight at 4°C, and permeabilized with 0.2% Triton X-100 in PBS for 10 min. Next, the cells were washed with PBS and 3% bovine serum albumin (blocking reagent) for 1 h. Afterwards, the cells were sequentially incubated with diluted primary antibodies (1:1000) for 1 h, washed thrice with wash buffer, and incubated with fluorescent secondary antibodies for 1 h in the dark. Finally, the coverslips were washed with wash buffer for analysis under confocal microscope.

### RNA isolation and quantitative real-time PCR (qRT-PCR)

The method was as described previously [26]. Briefly, total RNA was extracted by homogenization in 1 mL TRIZOL Reagent (Invitrogen), followed by chloroform extraction and isopropanol precipitation. The analysis of HIF-1α, VEGF, and IL-8 mRNA relative levels was performed using One Step SYBR PrimeScript RT-PCR (TaKaRa, China) according to the manufacturer’s instructions. A 50 ng sample of total RNA from A549 or NCI-H460 cells was used. The primers used were as follows: for human HIF-1α: forward 5′-TCTGGGTTGAAACTCAAGCAACTG-3′ and reverse 5′-CAACCGGTTTAAGGACACATTCTG-3′ [Genbank: NM_001243084.1]; VEGF: forward5′-TCTACCTCCACCATGCCAAGT-3′ and reverse 5′-GATGATTCTGCCCTCCTCCTT-3′ [Genbank: NM_001025366.2]; IL-8: forward 5′-TTGCCAAGGAGTGCTAAAGAA-3′ and reverse 5′-GCCCTC TTCAAAAACTTCTCC-3′ [Genbank: NM_000584.3]; β-actin: forward 5′-TCAAGATCATTGCTCCTCCTG-3′ and reverse 5′-CTGCTTGCTGATCCACATCTG-3′ [Genbank: NM_001017992.3]. All the primers were synthesized by TaKaRa Biotechnology (Dalian,) Co., LTD (Dalian, China). The thermocycling conditions were as follows: 42°C for 5 min, 95°C for 10 s, followed by 40 cycles at 95°C for 5 s, and 60°C for 31s. The relative HIF-1α, VEGF, and IL-8 mRNA levels were normalized to β-actin. The experiment was repeated in triplicate.

### ELISA

The concentration of VEGF and IL-8 in the conditioned media derived from treated and untreated cells was measured using ELISA reagent kits (Wuhan Boster Bio-engineering limited company, Wuhan, China) according to the manufacturer’s instructions. The experiment was repeated in triplicate.

### *In vitro* angiogenesis assay

An *in* *vitro* angiogenesis assay kit was employed according to the manufacturer’s instructions (Millipore). 96-well plates were coated with 50 µL of cold liquid ECMatrix each well and incubated at 37°C for 1 h to promote solidification. Afterwards, HUVECs were seeded at a density of 5×10^3^ cells/well into 96-well plates pre-coated with polymerized ECMatrix and incubated with conditioned media at 37°C for 6 to 8 h. The tubule formation was observed under a phase-contrast microscope, and the total tubule length in 3 random view-fields per well was measured by Scion image software and average value was calculated. The experiment was repeated in triplicate.

### Statistical analysis

For all experiments, data were presented as mean ± SD for three separate experiments. ANOVA and LSD-test were employed for statistical analysis using SPSS version 19.0. *P*<0.05 was considered to be statistically significant.

## Results

### HPV-16 E6 and E7 oncoproteins promoted the activation of PI3K/Akt/mTOR signaling pathway

**Figure 1:**
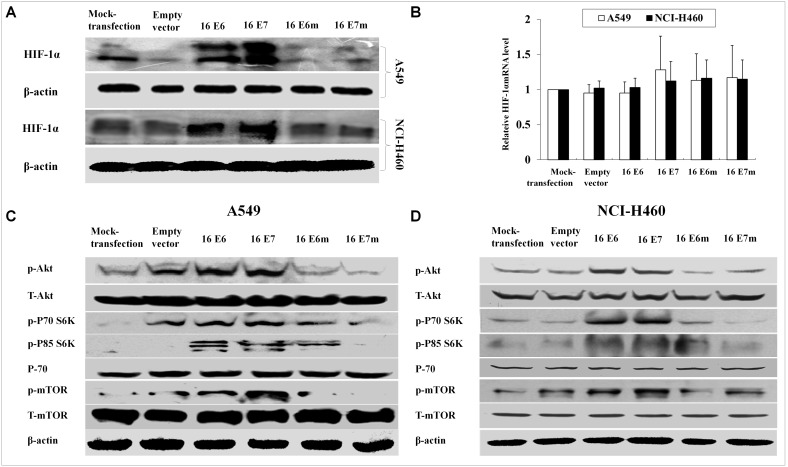
Effects of HPV-16 oncoproteins on HIF-1α expression and PI3K/Akt/mTOR signaling pathway activation in NSCLC cells. (A) Western blot analysis of HIF-1α protein levels in stable-transfected A549 cells. (B) Real-time PCR analysis of HIF-1α mRNA levels in stable-transfected A549 cells. (C and D) Western blot analysis of p-Akt, p-P70S6K, p-P85S6K, and p-mTOR protein levels in transfected A549 (C) and NCI-H460 (D) cells.

We previously established stable-transfected NSCLC cell lines (A549 and NCI-H460) using enhanced green fluorescent protein (EGFP) plasmid vectors harboring HPV-16 *E6* or *E7* gene, and the expression of HPV-16 E6 or E7 oncoprotein in the stable-transfected cells was confirmed [34]. HIF-1α protein and mRNA expression levels were analyzed in the stable-transfected A549 cells. Our results showed that HIF-1α protein levels were up-regulated by HPV-16 oncoproteins but HIF-1α mRNA levels had no significant difference (Figure 1A, B), which was the same as our previous results using transient transfection method [33]. PI3K/Akt/mTOR signaling pathway plays an important role in the development of NSCLC [31]. Moreover, our previous study has demonstrated that HPV-16 oncoproteins promoted the activation of PI3K/Akt in cervical cancer cells [33]. To investigate the effect of HPV-16 oncoproteins on PI3K/Akt/mTOR activation in NSCLC cells, we determined the phosphorylated levels of Akt, P70S6K, P85S6K, and mTOR in stable-transfected A549 cells. Our results showed that over-expression of HPV-16 E6 and E7 oncoproteins enhanced the phosphorylated levels of Akt, P70S6K, P85S6K, and mTOR in A549 NSCLC cells (Figure 1C). Similar results were found in another NSCLC cell line NCI-H460 (Figure 1D). These results indicated that HPV-16 oncoproteins activated PI3K/Akt/mTOR signaling pathway in NSCLC cells.

### PI3K/Akt signaling pathway was involved in HPV-16 E6- and E7-induced HIF-1α, VEGF, and IL-8 expression and *in* *vitro* angiogenesis

**Figure 2:**
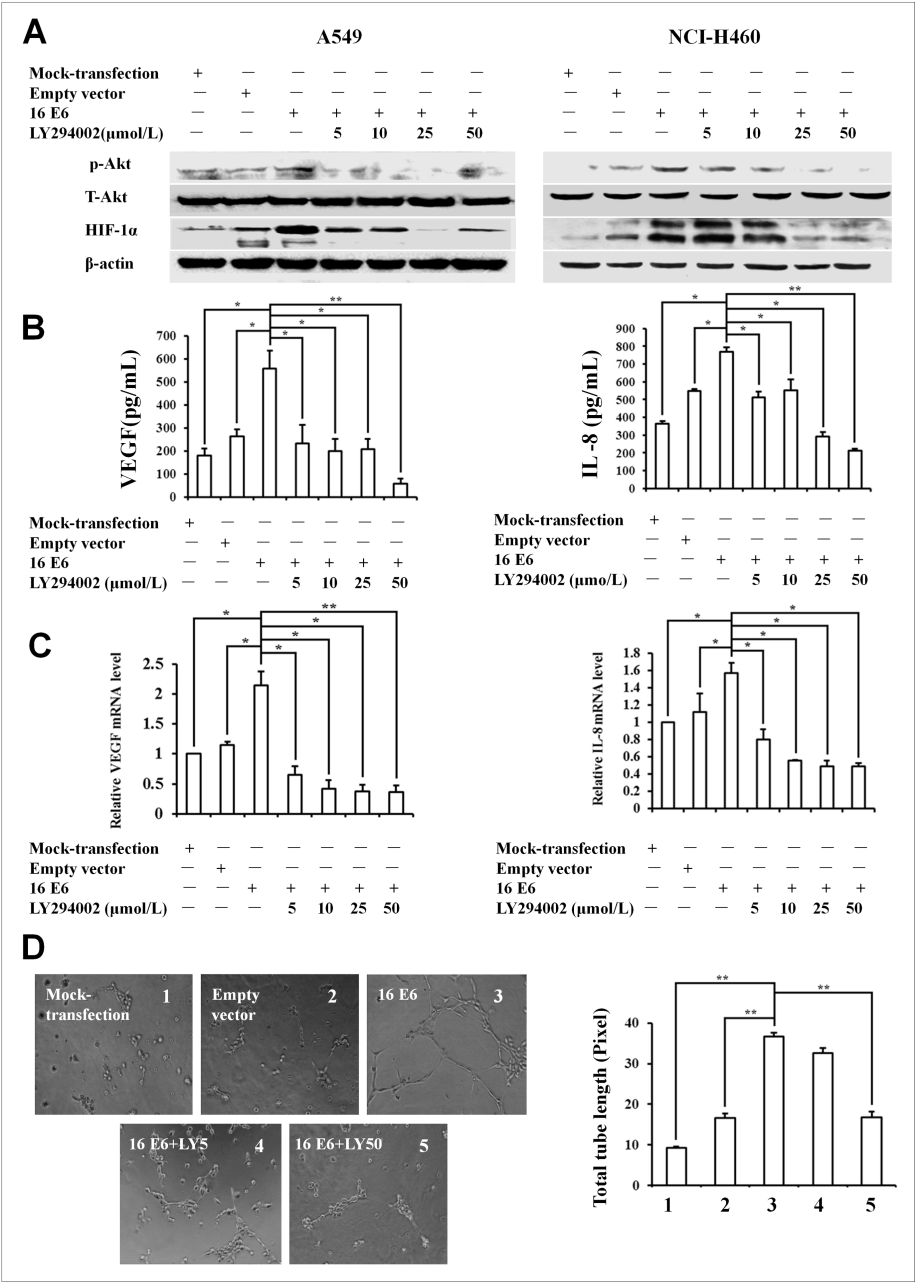
Effect of LY294002 on HPV-16 E6-induced HIF-1α, VEGF, and IL-8 expression and *in vitro* angiogenesis in NSCLC cells. HPV-16 E6-transfected NSCLC cells were pretreated for 24 h with different concentrations of LY294002. (A) HIF-1α and p-Akt protein levels in transfected NSCLC cells (Left: A549, Right: NCI-H460) were analyzed by Western blotting. (B) VEGF and IL-8 protein concentration in the conditioned media derived from transfected A549 cells was determined by ELISA. (C) VEGF and IL-8 mRNA levels in transfected A549 cells were determined by real-time PCR. (D) HUVECs (5×10^3^cells/well) were seeded onto the surface of 96-well cell culture plates pre-coated with polymerized ECMatrix and then incubated at 37°C for 6 to 8 h in the conditioned media derived from HPV-16 E6-transfected A549 cells in the absence or presence of LY294002. Left: The tube formation was observed under a phase-contrast microscope (20×). Right: The total tube length in three random view-fields per well was by Scion image software measured and average value was calculated. All data are expressed as mean ± SD of three independent experiments. *P<0.05, **P<0.01.

**Figure 3:**
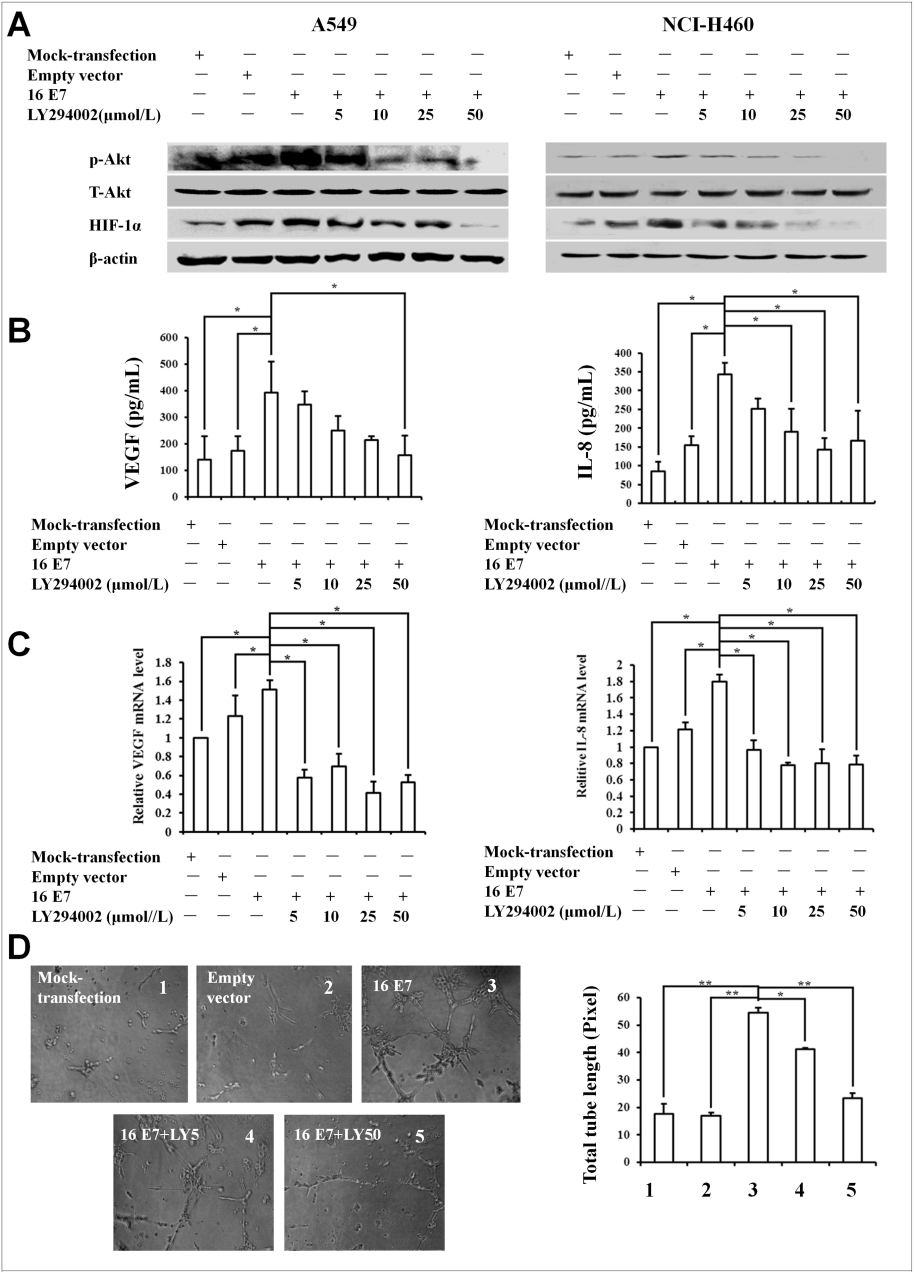
Effect of LY294002 on HPV-16 E7-induced HIF-1α, VEGF, and IL-8 expression and *in vitro* angiogenesis in NSCLC cells. HPV-16 E7-transfected NSCLC cells were pretreated for 24 h with different concentrations of LY294002. (A) HIF-1α and p-Akt protein levels in transfected NSCLC cells (Left: A549, Right: NCI-H460) were analyzed by Western blotting. (B) VEGF and IL-8 protein concentration in the conditioned media derived from transfected A549 cells was determined by ELISA. (C) VEGF and IL-8 mRNA levels in transfected A549 cells were determined by real-time PCR. (D) Results *in* *vitro* angiogenesis (A549 cells). HUVECs (5×10^3^cells/well) were seeded onto the surface of 96-well cell culture plates pre-coated with polymerized ECMatrix and then incubated at 37°C for 6 to 8 h in the conditioned media derived from HPV-16 E7-transfected A549 cells in the absence or presence of LY294002. Left: The tube formation was observed under a phase-contrast microscope (20×). Right: The total tube length in 3 random view-fields per well was by Scion image software measured and average value was calculated. All data are expressed as mean ± SD of three independent experiments. *P<0.05, **P<0.01.

The inhibition of PI3K activity has been reported to block HIF-1α activation and estrogen receptor recruitment to the VEGF promoter [36], [37]. Therefore, we further analyzed whether PI3K/Akt signaling pathway is involved in the expression of HIF-1α induced by HPV-16 oncoproteins in NSCLC cells. As shown in Figure 2 and 3, the pretreatment with different concentrations of LY294002, a specific PI3K inhibitor, significantly inhibited HPV16 E6- (Figure 2) and E7- (Figure 3) induced HIF-1α protein expression in both A549 and NCI-H460 cells (Figure 2A, 3A). Our previous study has shown that the expression of VEGF and IL-8 induced by HPV-16 oncoproteins in NSCLC cells is HIF-1α-dependent [26]. In this study, we further explored the role of PI3K/Akt signaling pathway in the expression of VEGF and IL-8 induced by HPV-16 E6 and E7 oncoproteins in NSCLC cells. Our results showed that the pretreatment with LY294002 remarkably inhibited HPV-16 oncoprotein-induced VEGF and IL-8 protein secretion (*P*<0.05, Figure 2B and 3B) and mRNA expression (*P*<0.05, Figure 2C and 3C) in both A549 and NCI-H460 cells. These findings suggested that PI3K/Akt signaling pathway was involved in HPV-16 E6- and E7-induced HIF-1α, VEGF, and IL-8 expression.

HIF-1α, VEGF, and IL-8 are important angiogenic factors. To deeply analyze the role of PI3K/Akt signaling pathway in angiogenesis, an *in* *vitro* angiogenesis model was employed to evaluate the capillary tube formation of HUVECs stimulated by the conditioned media derived from stable-transfected A549 cells. Our results showed that LY294002 significantly blocked HPV-16 oncoprotein-stimulated formation of capillary tube-like structures (*P*<0.05, Figure 2D and 3D), indicating that PI3K/Akt signaling pathway was involved in angiogenesis *in* *vitro* induced by HPV-16 oncoproteins.

### HPV-16 oncoproteins promoted HIF-1α protein stability *via* blocking 26S proteasome degradation pathway

**Figure 4:**
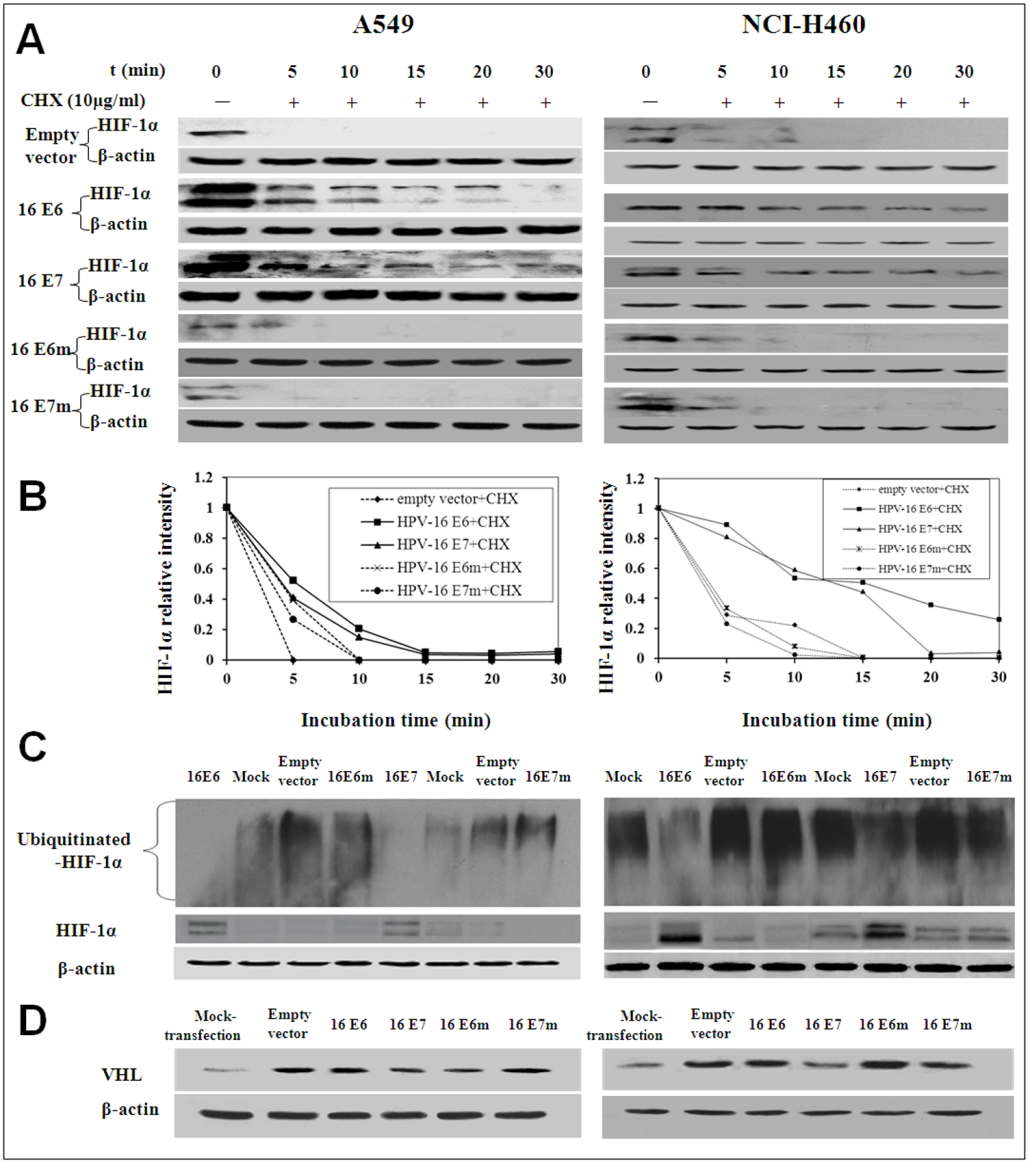
Stability of HIF-1α protein in HPV-16 E6 or E7-transfected NSCLC cells. (A) HPV-16 E6- or E7-transfected NSCLC cells (Left: A549, Right: NCI-H460) were treated with 10 µg/mL of cycloheximide (CHX) for different time periods. HIF-1α protein levels were determined by Western blotting. (B**)** Quantitative densitometric analysis of results from A**.** (C) HPV-16 E6- or E7-transfected NSCLC cells (Left: A549, Right: NCI-H460) were treated with 20 µmol/L of MG-132 for 6 h. Western blotting was performed to determine HIF-1α protein levels. (D) VHL protein levels in transfected NSCLC cells (Left: A549, Right: NCI-H460) were analyzed by Western blotting. Data presented are representative of results from three independent experiments.

Our results showed that HPV-16 E6 and E7 oncoproteins enhanced HIF-1α protein accumulation but had no effect on HIF-1α mRNA expression in A549 cells (Figure 1A and B). Our previous studies have demonstrated that HIF-1α protein accumulation induced by hypoxia or IGF-1 was through enhancing HIF-1α protein stability [28]–[30]. To analyze the effect of HPV-16 oncoproteins on HIF-1α protein stability, we used cycloheximide (CHX) to prevent further synthesis of HIF-1α protein in A549 and NCI-H460 cells. We found that HPV-16 E6 and E7 oncoproteins obviously inhibited HIF-1α protein degradation as compared with empty vector or mutant controls in A549 and NCI-H460 cells (Figure 4A and B), suggesting that HPV-16 E6 and E7 oncoproteins enhanced HPV-16 oncoprotein-induced HIF-1α protein expression possibly by inhibiting its degradation. To further explore whether HPV-16 oncoproteins inhibited HIF-1α degradation is through interfering with 26S proteasome degradation pathway, A549 and NCI-H460 cells were treated with MG132, a potent and specific 26S proteasome inhibitor. Our results showed that HPV-16 E6 and E7 oncoproteins decreased ubiquitinated HIF-1α levels in both A549 and NCI-H460 cells (Figure 4C), suggesting that HPV-16 oncoproteins promoted HIF-1α stability may be *via* blocking 26S proteasome degradation pathway. To deeply study the effect of HPV-16 oncoproteins on ubiquitination, we analyzed the protein expression of von Hippel-Lindau (VHL), an important component of the E3 ubiquitin ligase complex. Our results showed that HPV-16 E7 oncoprotein partly inhibited VHL protein expression as compared with empty vector or HPV-16 E7 mutant, but E6 oncoprotein had no obvious effect on VHL protein expression (Figure 4D).

### c-Jun was involved in HPV-16 oncoprotein-induced HIF-1α, VEGF, and IL-8 expression and *in* *vitro* angiogenesis

**Figure 5:**
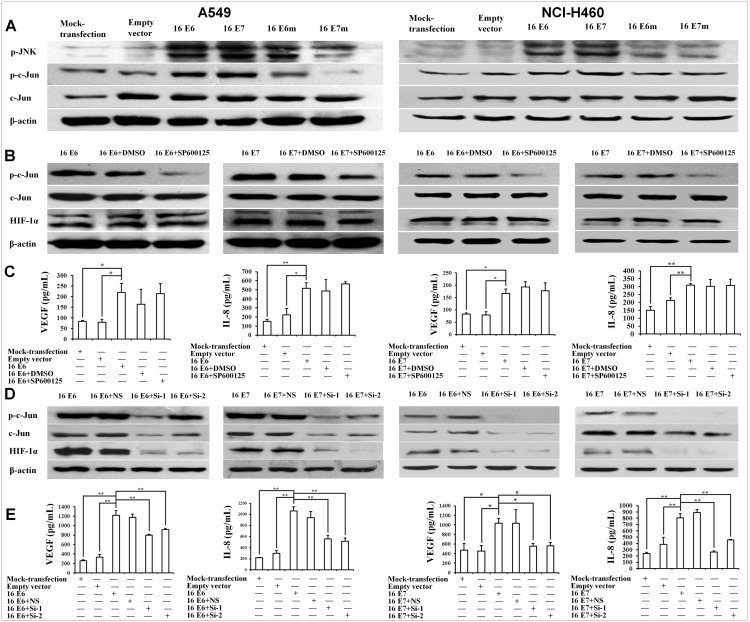
Role of JNK/c-Jun signaling pathway in HIF-1α, VEGF, and IL-8 expression induced by HPV-16 oncoproteins in NSCLC cells. (A) Western blot analysis of p-JNK, p-c-Jun, and c-Jun protein levels in stable-transfected NSCLC cells (Left: A549, Right: NCI-H460). (B and C) HPV-16 E6- or E7-transfected NSCLC cells (Left: A549, Right: NCI-H460) were pretreated with SP600125. HIF-1α, p-c-Jun, and c-Jun protein levels were analyzed by Western blotting (B), and VEGF and IL-8 protein concentration in the conditioned media was determined by ELISA (C). (D and E) HPV-16 E6- or E7-transfected NSCLC cells (Left: A549, Right: NCI-H460) were co-transfected with c-Jun siRNA (Si-1 or Si-2) or non-specific siRNA (NS-siRNA). HIF-1α, p-c-Jun, and c-Jun protein levels were analyzed by Western blotting (D), and VEGF and IL-8 protein concentration in the conditioned media was determined by ELISA (E). All data are expressed as mean ± SD of three independent experiments. *P<0.05, **P<0.01.

**Figure 6:**
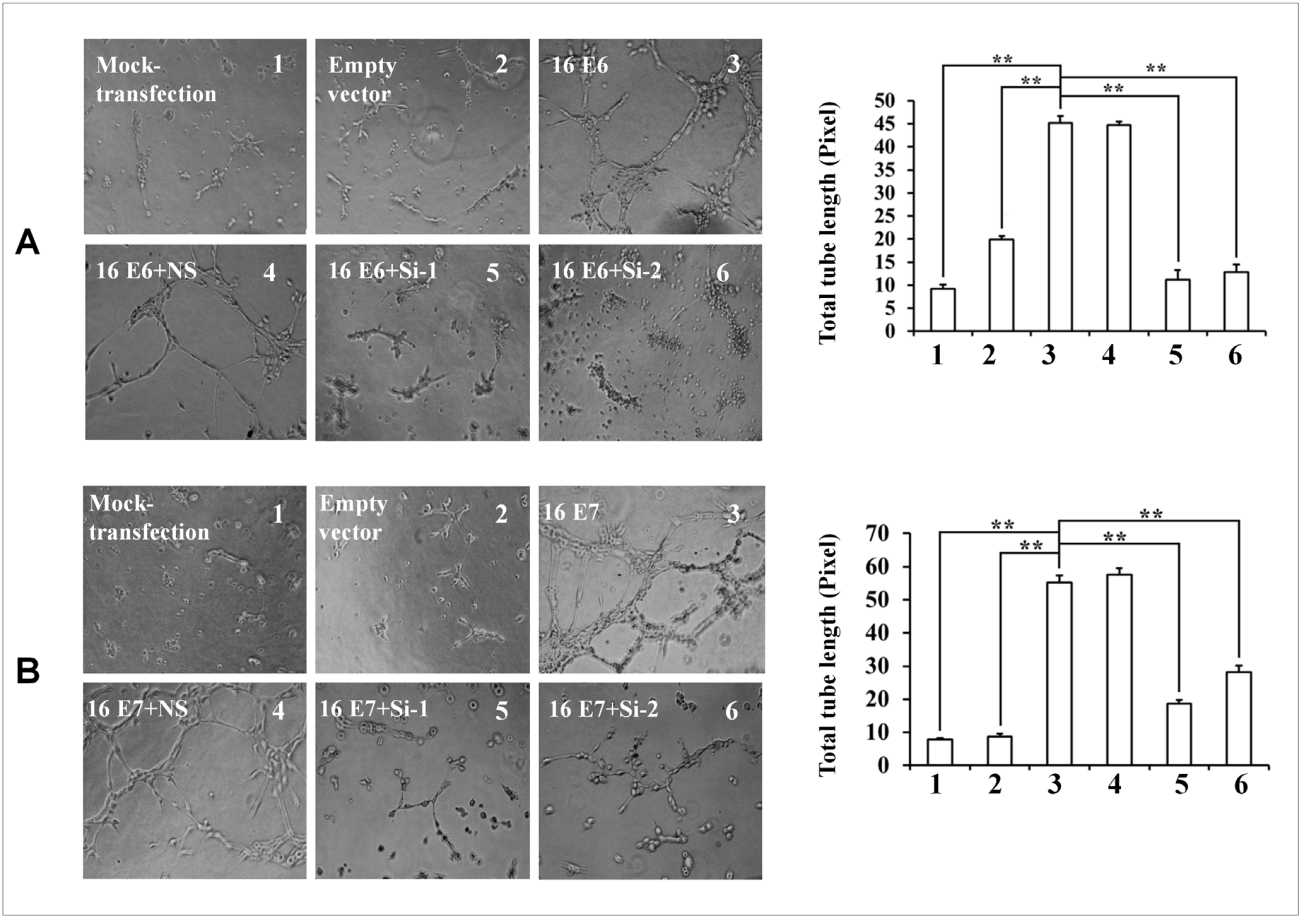
Effect of c-Jun siRNA on angiogenesis *in vitro* stimulated by over-expression of HPV-16 E6 or E7 in A549 cells. HPV-16 E6- (A) or E7- (B) transfected A549 cells were co-transfected with c-Jun siRNA (Si-1 or Si-2). Left: The tube formation was observed under a phase-contrast microscope (20×). Right: The total tube length in three random view-fields per well was by Scion image software measured and average value was calculated. All data are expressed as mean ± SD of three independent experiments. **P<0.01.

Previous studies have suggested that phosphorylated c-Jun (p-c-Jun) may be associated with HIF-1α degradation and accumulation [35]. To validate the role of p-c-Jun in HIF-1α degradation inhibited by HPV-16 oncoproteins in NSCLC cells, we analyzed p-c-Jun and its upstream protein JNK expression using Western blotting. The data confirmed that HPV-16 oncoproteins, especially E7 oncoprotein, enhanced p-c-Jun and p-JNK protein expression in A549 and NCI-H460 cells (Figure 5A), suggesting that JNK/c-Jun signaling pathway could be activated by HPV-16 oncoproteins. To further explore whether JNK/c-Jun signaling pathway is involved in HIF-1α protein accumulation induced by HPV-16 oncoproteins, two types of NSCLC cell lines were pretreated with SP600125, a specific JNK inhibitor, and HIF-1α protein expression was detected by Western blotting. The results showed that SP600125 down-regulated p-c-Jun protein levels, but HIF-1α protein levels had no significant changes when p-c-Jun was inhibited (Figure 5B). We also determined VEGF and IL-8 concentration in the conditional media derived from the cells pretreated with SP600125. We also found that SP600125 had no obvious effect on VEGF and IL-8 protein secretion induced by HPV-16 oncoproteins (Figure 5C). These results suggested that HIF-1α expression and HIF-1α-mediated VEGF and IL-8 expression were JNK-independent. To explore whether c-Jun makes a contribution to HPV-16 oncoprotein-induced HIF-1α, VEGF, and IL-8 expression, HPV-16-transfected NSCLC cells were co-transfected with c-Jun siRNA (Si-1 or Si-2). As shown in Figure 5D, HPV-16 oncoprotein-induced HIF-1α protein expression was down-regulated when c-Jun was inhibited by c-Jun siRNA. Expectantly, HPV-16 oncoprotein-induced VEGF and IL-8 protein secretion in NSCLC cells was also decreased in response to c-Jun knockdown (Figure 5E). Accordingly, we further found that c-Jun siRNAs (Si-1 and Si-2) dramatically inhibited angiogenesis *in* *vitro* stimulated by over-expression of HPV-16 E6 and E7 oncoproteins in A549 cells (Figure 6).

### HPV-16 oncoproteins promoted HIF-1α protein stability possibly through enhancing the interaction between c-Jun and HIF-1α

**Figure 7:**
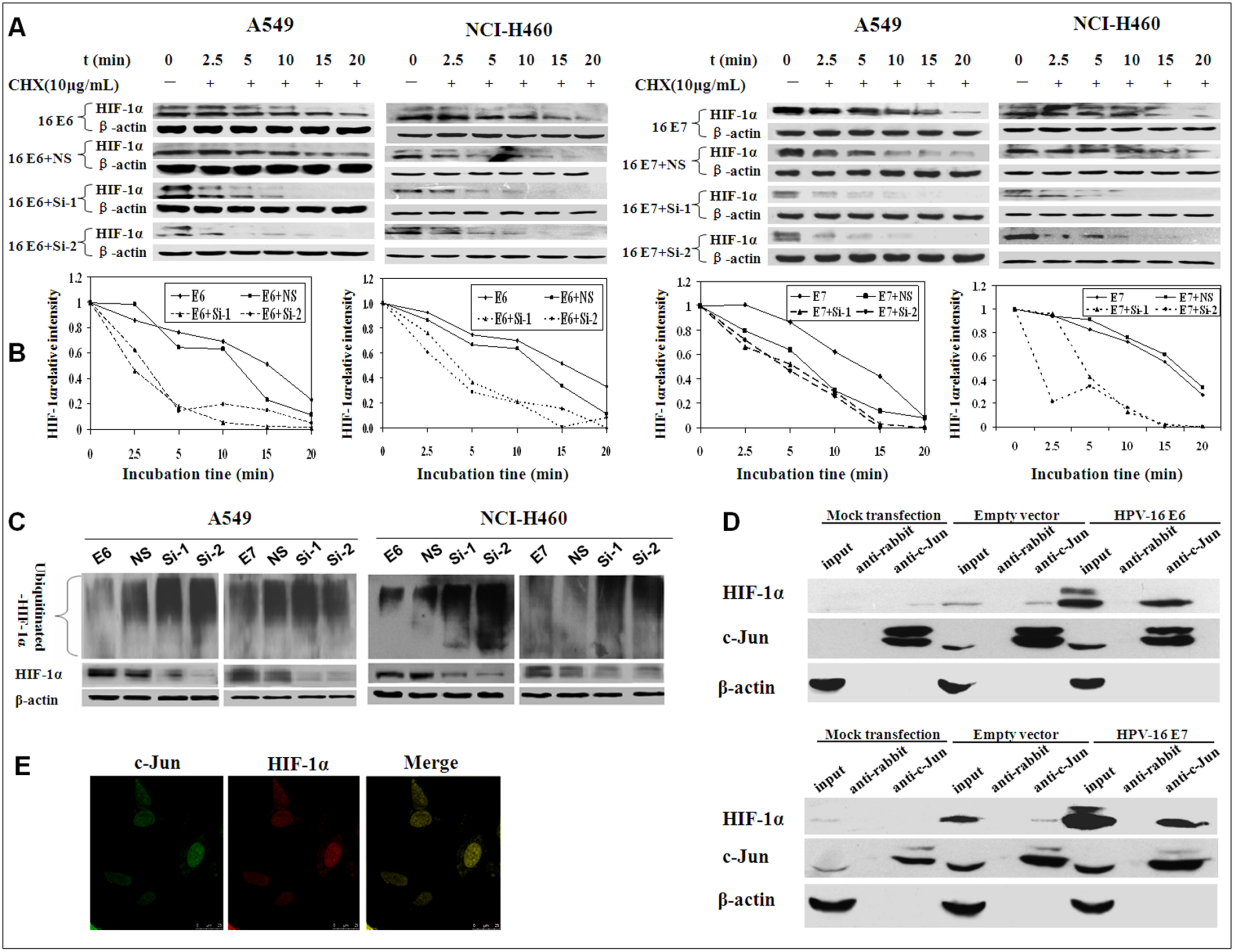
HPV-16 oncoproteins promoted HIF-1α protein stability through enhancing the interaction between c-Jun and HIF-1α. (A) HPV-16 E6-(Left) or E7-(Right) transfected A549 and NCI-H460 cells were co-transfected with c-Jun siRNA (Si-1 or Si-2) or NS-siRNA, followed by treatment with 10 µg/mL CHX for different time periods. HIF-1α protein levels were determined by Western blotting. (B) Quantitative densitometric analysis of results from A. (C) HPV-16 E6- or E7-transfected A549 (Left) and NCI-H460 cells (Right) were co-transfected with c-Jun siRNA (Si-1 or Si-2) or NS-siRNA, followed by treatment with 20 µmol/L of MG-132 for 6 h. Western blotting was performed to determine HIF-1α protein levels and ubiquitination. (D) Co-immunoprecipitation results of HIF-1α and c-Jun in A549 cells. Upper: HPV-16 E6; Lower: HPV-16 E7. (E) Cell immunofluorescence results in A549 cells (40×). The green area showed the position of c-Jun expression in A549 cells. The red area showed the position of HIF-1α expression in A549 cells. The yellow area showed the position of HIF-1α and c-Jun co-expression. Data presented are representative of results from three independent experiments.

To analyze the role of c-Jun in the stability of HIF-1α protein enhanced by HPV-16 oncoproteins, HPV-16-transfected NSCLC cells were co-transfected with c-Jun siRNA (Si-1 or Si-2) or nonspecific (NS)-siRNA, followed by treatment with 10 µg/mL CHX. Our results showed that the increased HIF-1α protein stability induced by HPV-16 oncoprotein was abrogated by c-Jun siRNA (Si-1 or Si-2) co-transfection, but not by NS-siRNA co-transfection (Figure 7A, B), indicating that HPV-16 oncoproteins enhanced HIF-1α protein stability in NSCLC cells was c-Jun-dependent. To further examine whether the role of c-Jun is *via* interfering with 26S proteasome-dependent degradation pathway, HPV-16-transfected NSCLC cells were co-transfected with c-Jun siRNA (Si-1 or Si-2) or NS-siRNA, followed by treatment with MG132. As shown in Figure 7C, the ubiquitination of HIF-1α protein was increased in HPV-16- and c-Jun siRNA-co-transfected cells as compared with controls. Taken together, these findings suggested that c-Jun may play an important role in the enhancement of HIF-1α protein stability induced by HPV-16 oncoproteins *via* blocking 26S proteasome-dependent degradation pathway in NSCLC cells.

Previous studies have demonstrated that c-Jun can interact with HIF-1α *via* oxygen-dependent degradation (ODD) domain, thus preventing HIF-1α from 26S proteasome-dependent degradation [35]. According to our results and previous studies, we hypothesize that HPV-16 oncoproteins inhibit HIF-1α protein degradation *via* enhancing the interaction between HIF-1α and c-Jun. To verify the hypothesis, co-immunoprecipitation was performed to determine the quantity of c-Jun binding to HIF-1α. As shown in Figure 7D, HPV-16 E6 and E7 oncoproteins obviously increased the quantity of c-Jun-HIF-1α complex in A549 cells. Furthermore, the results from cell immunofluorescence showed that c-Jun and HIF-1α proteins were co-localized in the nuclei (Figure 7E). Therefore, our results confirmed the hypothesis.

## Discussion

Accumulating evidence has demonstrated that HPV-16 oncoproteins can promote angiogenesis by up-regulating the expression of a variety of pro-angiogenic factors including fibroblast growth factor binding protein, basic fibroblast growth factor, transforming growth factor-β, tumor necrosis factor-α, angiopoietin-1, hepatocyte growth factor, and placental growth factor in cervical cancer cells [33], [38]–[40]. Our previous studies have also found that HPV-16 E6 and E7 oncoproteins enhanced angiogenesis by up-regulating HIF-1α protein accumulation and HIF-1α-dependent VEGF and IL-8 expression in NSCLC cells [26], but the underlying mechanisms are not known. An increasing body of evidence has demonstrated that multiple signaling pathways including PI3K/Akt and JNK/c-Jun are involved in the up-regulation of HIF-1α, VEGF, and IL-8 expression stimulated by different factors [27]–[30]. In this study, we first demonstrated that the roles of PI3K/Akt and c-Jun in the expression of HIF-1α, VEGF, and IL-8 stimulated by HPV-16 oncoproteins in NSCLC cells.

PI3K/Akt signaling pathway is well known to play a key role in regulating angiogenesis in various cancers including NSCLC [31], [41], [42]. Especially, our previous study has demonstrated that HPV-16 E6 and E7 induced HIF-1α protein accumulation and VEGF expression *via* PI3K/Akt signaling pathway in human cervical cancer cells [33]. In the present study, we also detect the effect of HPV-16 E6 and E7 oncoproteins on the activation of PI3K/Akt signaling pathway in NSCLC cells. We found that HPV-16 E6 and E7 oncoproteins activated PI3K/Akt signaling pathway in two types of NSCLC cell lines, A549 and NCI-H460 cells. Moreover, the expression of HIF-1α, VEGF, and IL-8 and angiogenesis *in* *vitro* were inhibited by LY294002, a specific PI3K inhibitor. Taken together, our results suggest that PI3K/Akt signaling pathway may be involved in the expression of HIF-1α, VEGF, and IL-8 induced by HPV-16 E6 and E7 oncoproteins in NSCLC cells, leading to angiogenesis *in* *vitro*. However, our results from Western blotting showed that the highest dose of LY294002 reversed HIF-1α protein expression, but VEGF expression was not consistent with the data of western blotting, which could reflect the possibility that PI3K/Akt signaling pathway may be not fully responsible for the expression of HIF-1α, VEGF, and IL-8.

mTOR, the downstream effector of Akt, assembles into two complexes with distinct inputs and downstream effects: mTOR complex 1 (mTORC1) and mTORC2. Akt activity can lead to the activation of mTORC1, an important regulator of cellular growth and protein synthesis [42]. Under favorable growth conditions, activated mTOR regulates the phosphorylation of its downstream targets, eIF4E-binding protein 1 (4E-BP1) and S6 kinase (S6K), resulting in protein translation initiation and elongation. Interestingly, in this study, we found that over-expression of HPV-16 E6 and E7 oncoproteins activated mTOR, P70S6K, and P85S6K in NSCLC cells. Therefore, whether mTOR signaling pathway mediated HPV-16 oncoprotein-induced HIF-1α, VEGF, and IL-8 expression in NSCLC cells is worthy of further investigation.

HIF-1α, an upstream regulator of VEGF, triggers angiogenesis through VEGF in various types of cancer. HIF-1α expression is highly regulated by oxygen concentration. Under normoxic conditions, HIF-1α is hydroxylated in its oxygen-dependent degradation domain by propyl hydroxylases (PHD) [43]. Afterwards, hydroxylated HIF-1α is recognized by a protein complex combining with VHL, leading to poly-ubiquitination and degradation. Under hypoxic conditions, HIF-1α is unable to be hydroxylated by PHD, allowing it to escape from ubiquitination and degradation. Besides hypoxia, other non-hypoxic factors can also be found to up-regulate HIF-1α protein levels [28]–[30], [33]. In our previous and present studies, we found that over-expression of HPV-16 E6 and E7 oncoproteins up-regulated HIF-1α protein expression both in transiently [26] and stably transfected NSCLC cells (Figure 1A). HPV-16 E6 and E7 oncoproteins seem to create the same “protein microenvironment” like hypoxic conditions to up-regulate HIF-1α protein levels. However, our previous and present results showed that over-expression of HPV-16 E6 and E7 had no effect on HIF-1α mRNA expression in NSCLC cells [26], indicating that HPV-16 oncoproteins enhanced HIF-1α protein accumulation *via* a post-transcriptional mechanism, *e.g.* affecting HIF-1α protein stability. As expected, in this study, our results showed that HPV-16 E6 and E7 oncoproteins significantly suppressed HIF-1α protein degradation in NSCLC cells (Figure 4A, B). Moreover, we further found that HPV-16 E6 and E7 oncoproteins decreased ubiquitinated HIF-1α levels in NSCLC cells (Figure 4C), suggesting that HPV-16 oncoproteins inhibited HIF-1α degradation is possibly, at least in part, through interfering with 26S proteasome degradation pathway, thus triggering HIF-1α protein accumulation in NSCLC cells. VHL, the substrate recognition subunit of an E3 ligase, is well known to contribute to HIF-1α degradation. However, in this study, we found that over-expression of HPV-16 oncoproteins, especially E6, had no obvious effect on VHL protein expression, indicating that HPV-16 E6 oncoprotein inhibited HIF-1α degradation *via* VHL-independent pathways and the other underlying mechanisms possibly contribute to HIF-1α protein stability mediated by HPV-16 E6 oncoprotein in NSCLC cells.

c-Jun and its upstream protein JNK have long been considered to be associated with angiogenesis. When c-Jun was suppressed in human endothelial cells, the cells no longer form new blood vessels *in* *vitro* or *in* *vivo* [44]. Additionally, c-Jun is an essential for high production of VEGF under hypoxic conditions, and the involvement of c-Jun can enhance VEGF transcription [45], [46]. Our results showed that when c-Jun was inhibited by its specific siRNA, the increase of HIF-1α, VEGF, and IL-8 protein expression (Figure 5D and E) induced by HPV-16 oncoproteins in NSCLC cells was also blocked (Figure 5D and E), suggesting that HIF-1α, VEGF, and IL-8 protein expression induced by HPV-16 oncoproteins was c-Jun-dependent. Furthermore, we also found the knockdown of c-Jun remarkably inhibited HPV-16 E6 and E7 oncoprotein-stimulated angiogenesis *in* *vitro* in A549 cells (Figure 6). These data indicated that c-Jun-mediated pathway may be, at least in part, involved in HPV-16 oncoprotein-induced HIF-1α, VEGF, and IL-8 expression in NSCLC cells, thus leading to angiogenesis *in* *vitro*.

Generally, the phosphorylation of c-Jun at serine^63/73^ activates c-Jun-dependent transcription. Moreover, previous reports have demonstrated that phosphorylated c-Jun (p-c-Jun) binds to the VEGF promoter and regulates VEGF transcription directly [47]. Our results showed that the levels of p-c-Jun and its upstream p-JNK protein in NSCLC cells were up-regulated by HPV-16 oncoproteins, especially E7 (Figure 5A). However, the decease of p-c-Jun levels by SP600125, a specific JNK inhibitor, had no obvious effects on HIF-1α protein expression in HPV-16-transfected NSCLC cells (Figure 5B and C), suggesting HIF-1α protein accumulation induced by HPV-16 oncoproteins was JNK/c-Jun-independent in NSCLC cells. To explore whether c-Jun can mediate HPV-16 oncoprotein-induced HIF-1α protein accumulation in NSCLC cells *via* other pathways, the effect of c-Jun on HIF-1α protein stability was analyzed. Our results showed that c-Jun enhanced HIF-1α protein stability by inhibiting its degradation through 26S proteasome-dependent ubiquitination pathway (Figure 7A–C). Regularly, the reduction of VHL protein levels can decrease HIF-1α ubiquitination. However, our results showed that VHL protein levels had no significant changes in HPV-16 E6-transfected cells. Previous studies have demonstrated that c-Jun can interact with HIF-1α through ODD domain, thus preventing HIF-1α from 26S proteasome-dependent degradation [35]. Therefore, according to previous reports and our results, it can be hypothesized that HPV-16 oncoproteins may create a sort of “protein microenvironment” that can promote the combination between c-Jun and HIF-1α proteins and block the combination between VHL and HIF-1α ODD domain, leading to the inhibition of HIF-1α protein degradation. Interestingly, our results from co-immunoprecipitation and cell immunofluorescence verified this hypothesis. Our results showed that HPV-16 E6 and E7 oncoproteins obviously increased the quantity of c-Jun-HIF-1α complex in A549 cells (Figure 7D). Furthermore, c-Jun and HIF-1α proteins were co-localized in the nuclei (Figure 7E). Taken together, our findings suggest that HPV-16 E6 and E7 oncoproteins may inhibit HIF-1α protein degradation *via* enhancing the interaction between HIF-1α and c-Jun, thus contributing to HIF-1α-mediated angiogenesis in NSCLC.

In this study, we did not make further research on the relationships between PI3K/Akt and c-Jun signaling pathways. Different signaling pathways have a complex cross-talk network in cells. It may be a coordination between PI3K/Akt signaling pathway and c-Jun, along with other signaling pathways, thus making a contribution to HPV-16 oncoprotein-induced angiogenesis in NSCLC, which needs to be further studied.

## Conclusions

In this study, we demonstrated to our knowledge for the first time that PI3K/Akt signaling pathway and c-Jun are involved in HPV-16 oncoprotein-induced HIF-1α protein accumulation and VEGF and IL-8 expression in NSCLC cells. Moreover, HPV-16 oncoproteins promoted HIF-1α protein stabilization possibly by enhancing the interaction between c-Jun and HIF-1α, thus contributing to angiogenesis.
