# Correction: Roles of PI3K/Akt and c-Jun Signaling Pathways in Human Papillomavirus Type 16 Oncoprotein-Induced HIF-1α, VEGF, and IL-8 Expression and *In Vitro* Angiogenesis in Non-Small Cell Lung Cancer Cells

**DOI:** 10.1371/journal.pone.0314378

**Published:** 2024-11-20

**Authors:** Erying Zhang, Xiaowei Feng, Fei Liu, Peihua Zhang, Jie Liang, Xudong Tang

In Fig 2D, the image of 16 E6 is incorrect. Please see the correct Fig 2 here.

**Fig 2:**
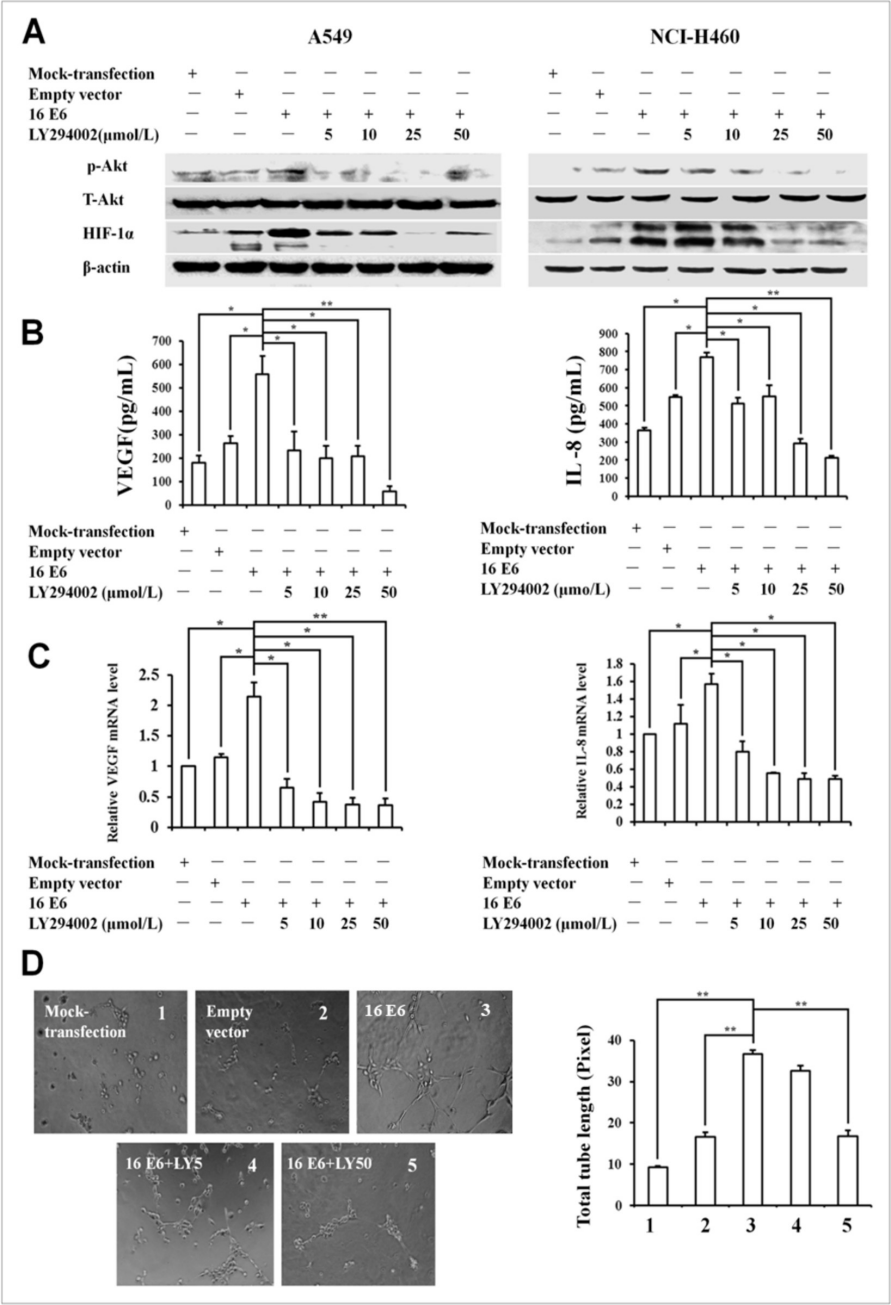
Effect of LY294002 on HPV-16 E6-induced HIF-1α, VEGF, and IL-8 expression and in vitro angiogenesis in NSCLC cells. HPV-16 E6-transfected NSCLC cells were pretreated for 24 h with different concentrations of LY294002. (A) HIF-1α and p-Akt protein levels in transfected NSCLC cells (Left: A549, Right: NCI-H460) were analyzed by Western blotting. (B) VEGF and IL-8 protein concentration in the conditioned media derived from transfected A549 cells was determined by ELISA. (C) VEGF and IL-8 mRNA levels in transfected A549 cells were determined by real-time PCR. (D) HUVECs (5×103 cells/well) were seeded onto the surface of 96-well cell culture plates pre-coated with polymerized ECMatrix and then incubated at 37°C for 6 to 8 h in the conditioned media derived from HPV-16 E6-transfected A549 cells in the absence or presence of LY294002. Left: The tube formation was observed under a phase-contrast microscope (20×). Right: The total tube length in three random view-fields per well was by Scion image software measured and average value was calculated. All data are expressed as mean ± SD of three independent experiments. *P<0.05,**P<0.01.
